# Genetic risk for Alzheimer’s dementia predicts motor deficits through multi-omic systems in older adults

**DOI:** 10.1038/s41398-019-0577-4

**Published:** 2019-10-03

**Authors:** Shinya Tasaki, Chris Gaiteri, Vladislav A. Petyuk, Katherine D. Blizinsky, Philip L. De Jager, Aron S. Buchman, David A. Bennett

**Affiliations:** 1 Rush Alzheimer’s Disease Center, Rush University Medical Center, Chicago, IL USA; 2 Department of Neurological Sciences, Rush University Medical Center, Chicago, IL USA; 3 Biological Sciences Division, Pacific Northwest National Laboratory, Richland, WA USA; 4 Center for Translational and Computational Neuroimmunology, Columbia University Medical Center, New York, NY USA; 5 Cell Circuits Program, Broad Institute, Cambridge, MA USA

**Keywords:** Clinical genetics, Clinical genetics, Epigenetics and behaviour, Molecular neuroscience, Diseases

## Abstract

Alzheimer’s disease manifests with both cognitive and motor deficits. However, the degree to which genetic risk of Alzheimer’s dementia contributes to late-life motor impairment, and the specific molecular systems underlying these associations, are uncertain. Here, we adopted an integrative multi-omic approach to assess genetic influence on motor impairment in older adults and identified key molecular pathways that may mediate this risk. We built a polygenic risk score for clinical diagnosis of Alzheimer’s dementia (AD-PRS) and examined its relationship to several motor phenotypes in 1885 older individuals from two longitudinal aging cohorts. We found that AD-PRS was associated with a previously validated composite motor scores and their components. The major genetic risk factor for sporadic Alzheimer’s dementia, the *APOE*/*TOMM40* locus, was not a major driver of these associations. To identify specific molecular features that potentially medicate the genetic risk into motor dysfunction, we examined brain multi-omics, including transcriptome, DNA methylation, histone acetylation (H3K9AC), and targeted proteomics, as well as diverse neuropathologies. We found that a small number of factors account for the majority of the influence of AD-PRS on motor function, which comprises paired helical filament tau-tangle density, H3K9AC in specific chromosomal regions encoding genes involved in neuromuscular process. These multi-omic factors have the potential to elucidate key molecular mechanisms developing motor impairment in the context of Alzheimer’s dementia.

## Introduction

There is increasing evidence that Alzheimer’s disease (AD) is implicated in late-life impairments in both cognitive and motor function^1–3^. This indicates that Alzheimer’s dementia and motor impairments in older adults may share a common underlying neurobiology. In prior work, we have shown relationships between AD and other brain pathologies and several different motor phenotypes^4–9^, but the genetic and molecular mechanisms underlying these associations are unknown.

Genetic variation has a significant impact on the development of Alzheimer’s dementia and cognitive decline^10–12^. While a few genetic variants associated with Alzheimer’s dementia, including variants in the ApoE^13^ and presenilin 1^14^ regions, have also been linked to motor deficits, the overall effect of AD genetics on motor impairment in older adults is yet to be examined in detail. Genetic findings could be linked to intermediate phenotypes, such as cell types^15^, canonical^16^, and data-driven pathways^17^, all of which aid in understanding Alzheimer’s dementia pathogenesis. Therefore, quantifying the genetic influence on motor impairment, and integration of molecular information, may be a useful avenue toward extracting actionable molecular mechanisms related to both cognitive and motor decline in older adults. Expected challenges to a genetic approach to motor function in older adults include the complex genetic basis of sporadic AD and logistical challenges of measuring motor phenotypes in large number of older individuals. Ideally, brain biospecimens would also be available from these individuals in order to extract molecular systems that mediate genetic risk for Alzheimer’s-related motor impairment.

**Fig. 1:**
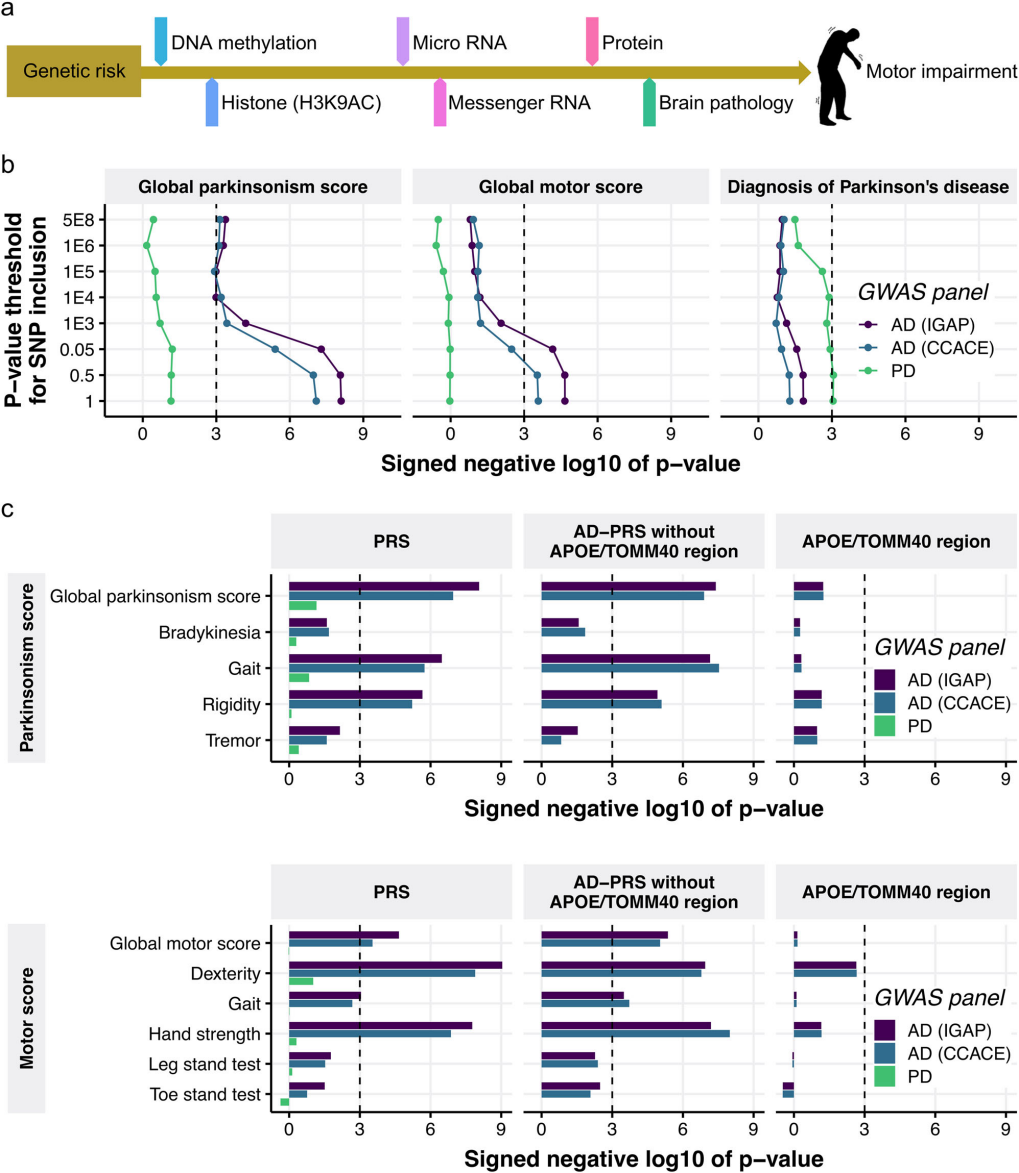
The genetic risk for Alzheimer’s dementia is associated with motor phenotypes. **a** Overview of this study. **b** Association of global motor scores and clinical diagnosis with PRSs with varying *p* value criteria for SNP inclusion. The sign of negative log 10 of *p* value indicates the direction of effect. **c** Association of motor domains with PRSs with the SNP inclusion *p* value of 0.5. For the AD-PRSs, the PRS without ApoE/TOMM40 SNPs and the PRS using only ApoE/TOMM40 SNPs were also tested. The sign of negative log 10 of *p* value indicates the direction of effect

In the present study, we examined relationships between genetic risk variants for Alzheimer’s dementia and motor function in older adults by aggregating genetic variants into a total risk score that is calculated for each individual (polygenic risk score, PRS). We tested if the PRS for Alzheimer’s dementia (AD-PRS) has any observable relationship to motor function (Fig. 1a). Then, we searched for a wide range of molecular and neuropathological features that might link the AD-PRS to motor function in older adults (Fig. 1a). These factors could serve as starting points for ex vivo and animal model investigations on the molecular mechanism underlying late-life motor impairment, supported by diverse genetic information and direct tests of motor function in thousands of older adults.

## Methods

### Cohort summaries for the Religious Orders Study (ROS) and Rush Memory and Aging Project (MAP)

The ROS and MAP studies, based out of the Rush Alzheimer’s Disease Center (RADC) in Chicago, are two longitudinal, community-based aging studies with many harmonized data measures, collectively referred to as ROSMAP^18^. Together, these ongoing studies have enrolled >3600 older persons, all of whom have agreed to brain donation and annual detailed clinical evaluation, including motor testing. As of March 2018, a total of 1885 individuals had completed genotyping and completed motor assessments. Almost all were non-Latino whites (99.4%) (Supplementary Table 1). Brain autopsies were reviewed and approved by a board-certified neuropathologist. All omics analyses except genotyping were performed on the dorsolateral prefrontal cortex (DLPFC)^19^. All phenotypes and omics data are shared widely with a data use agreement through the RADC Resource Sharing Hub (www.radc.rush.edu).

### Standard protocol approvals, registrations, and patient consents

The parent cohort studies and sub-studies were approved by Rush University Medical Center Institutional Review Board. All participants provided written informed consent and signed an Anatomic Gift Act for brain donation.

### Clinical diagnoses

Clinical cognitive diagnoses, including Alzheimer’s dementia, were based on criteria of the joint working group of the National Institute of Neurological and Communicative Disorders and Stroke and the Alzheimer’s Disease and Related Disorders Association (NINCDS/ADRDA). Diagnosis of mild cognitive impairment (MCI) was rendered for individuals who had cognitive impairment but did not meet criteria for dementia. Individuals without dementia or MCI were categorized as no cognitive impairment. Clinical diagnosis of Parkinson’s disease (PD) was made by a clinician through review of self-reported history, neurological exam, cognitive testing, and review of medications for PD therapies, including levodopa or dopamine agonists, based on clinical criteria recommended by the Core Assessment Program for Intracerebral Transplantation^9^. We set PD variable to 1 when an individual was diagnosed with PD and to 0 in individuals without PD.

### Assessment of motor function

Motor function is a complex behavior that may require several clinical instruments to capture the diverse deficits, which manifest in older adults. We examined two related phenotypes that we have validated in prior studies and that are independently associated with adverse health outcomes when considered together^8^.

#### Global parkinsonism score

There is increasing recognition that mild parkinsonian signs, including bradykinesia, tremor, rigidity, and parkinsonian gait, are also common in older adults without PD^4,9^. A modified version of the United Parkinson’s Disease Rating Scale was administered by trained nurse clinicians^20,21^. Twenty-six items were examined to assess four parkinsonian signs (parkinsonian gait, bradykinesia, rigidity, and tremor)^18^. Each sign was scored from 0 to 100, and a global parkinsonism score was formed by averaging the scores across the four individual parkinsonian domains. The global parkinsonism score and parkinsonian gait were square root transformed. Bradykinesia and rigidity were dichotomized and set to 0 if the sign was absent and to 1 when present. Higher score indicates more severe parkinsonian impairment of motor function.

#### Global motor score

Aging is associated with a wide spectrum of progressive motor dysfunction, including mild decreased muscle strength, impaired balance, and reduced speed and dexterity. Ten motor performances were assessed to adequately sample motor function across this spectrum. (1) Grip and (2) pinch strength were measured bilaterally using the Jamar® hydraulic hand and pinch dynamometers (Lafayette Instruments, Lafayette) to assess manual strength. Upper extremity dexterity was based on (3) the number of pegs that could be placed (Purdue Pegboard) in 30 s. Two trials were recorded for each hand. The four trials were averaged to provide a Purdue Pegboard score. To evaluate dexterity, (4) participants tapped an electronic tapper (Western Psychological Services, Los Angeles, CA) with their index finger as quickly as possible for 10 s. Two trials were performed for each hand. The four trials were averaged together to yield a tapping score. To evaluate gait, we asked people to walk eight feet and turn 360° and measured the (5, 6) time and (7, 8) number of steps taken on each task. (9) To assess balance, we asked people to stand on each leg for 10 s and the time that the participant was able to balance was recorded. (10) Persons were asked to stand on their toes for 10 s and the time that participant was able to perform the task was recorded^3,8^. All ten measures were scaled and averaged to obtain a summary global motor score, previously reported to be associated with risk of mortality, incident disability, and dementia^8,22^. ﻿Summary measures of manual strength (two tests), manual dexterity (two tests), and gait (four tests), were formed in a similar manner. We did not form a balance measure because the balance tests, unlike the other motor tests, were sometimes not attempted^3,22^. To maintain consistency with global parkinsonism score, the sign of global motor score and its components were reversed so that a higher score indicates more severe motor impairment.

### Assessment of neuropathology indices

We generated continuous measures for neuritic plaques, diffuse plaques, neurofibrillary tangles, the load of parenchymal deposition of β-amyloid, and the density of abnormally phosphorylated paired helical filament tau (PHFtau)-positive neurofibrillary tangles, as previously described^23–27^. We also quantified nigral neuronal loss^7^, Lewy bodies^28^, TDP-43 staging^29^, hippocampal sclerosis^30^, chronic macroscopic and microinfarcts infarcts^31^, cerebral amyloid angiopathy^32^, and severity of atherosclerosis^33^. Details on neuropathology indices were described in the Supplementary Methods and the complete list of brain pathologies assessed in this study is in Supplementary Table 2.

### Omics measurements

We generated genotyping data for 7,159,943 single-nucleotide polymorphisms (SNPs) in 2093 subjects from from peripheral blood mononuclear cells or frozen brain tissue^34,35^. We also quantified DNA methylation levels at ~130,000 loci^36^, levels of histone H3 acetylation on lysine 9 (H3K9AC) at 26,384 genomic regions^37^, expression of 13,484 genes^17,38^, expression of 292 microRNAs (miRNAs)^35,39^, and abundance of 67 proteins^40^ from DLPFC. To alleviate a large multiple testing burden, we followed the standard practice of reducing DNA methylation, histone acetylation, and gene expression to 58 comethylated, 80 coacetylated, and 49 coexpressed modules^41,42^. Details on omics measurements were described in the Supplementary Methods and the complete list of omics variables assessed in this study is in Supplementary Table 2.

### PRS generation

The genetic variants comprising our AD-PRS were identified based on genome-wide association study (GWAS) data from the International Genomics of Alzheimer’s Project (IGAP)^43^ and GWAS for family history of AD from the Center of Cognitive Ageing and Cognitive Epidemiology (CCACE)^44^. The IGAP study conducted a two-stage meta-analysis of 25,580 Alzheimer’s dementia cases and 48,466 controls. To select SNPs used in a PRS, we used the summary statistics of 11,632 SNPs from the entire cohort and those of three SNPs (rs769449, rs769450, and rs429358) located in the apolipoprotein E (*ApoE*) and translocase of outer mitochondrial membrane 40 (*TOMM40*) region from the first IGAP stage. For CCACE, the summary statistics of 8503 SNPs from the meta-analysis of IGAP and GWAS for family history of AD from 314,278 participants from the UK Biobank (27,696 maternal cases, 14,338 paternal cases) was used. Since 1072 subjects in these GWAS are part of the ROSMAP cohorts, we adjusted the summary statistics by subtracting the signals originated from ROSMAP participants as previously described^45^. To contrast with AD-PRS, we also built a PRS for PD (PD-PRS) based on the summary statistics from the largest recent meta-analysis (PDWBS)^46^. SNPs in linkage disequilibrium (LD) were pruned using the PRSice-2 software^47^ with the threshold of *R*^2^ > 0.1 and the window of 2000 kb using LD estimates based on all 2093 genotyped ROSMAP participants. This resulted in 1215, 1197, and 399 independent SNPs from IGAP, CCACE, and PDWBS, respectively (Supplementary Table 3). We calculated PRSs for 1885 individuals as an average of the number of risk-increasing allele weighted by the summary statistic (*β* or log odds) using the PRSice-2 software^47^. An AD-PRS without SNPs located in ApoE/TOMM40 region and an AD-PRS consisting of ApoE/TOMM40 SNPs were also generated in the same procedure as above. We then scaled PRSs by subtracting the mean PRSs across all individuals and dividing by the standard deviation.

### Statistical analysis

Linear or logistic regression models were used for testing association for a continuous or categorical outcome, respectively. The following variables were removed from the continuous outcome using linear regression leaving the residuals for use in the association test: age at measurement, sex, years of education, and the first three genotyping principal components (PCs) to account potential population stratification. For categorical outcomes, we fitted the logistic regression of the categorical outcome on age at measurement, sex, years of education, and the first three genotyping PCs. Obtained coefficients were kept constant in all subsequent models for that outcome. These constant terms for covariates are referred to as “offset” terms in the generalized linear model.

### Other bioinformatic analyses

To examine biological function of histone coacetylation, we performed gene ontology (GO) enrichment analysis^48–51^. To evaluate the proportion of AD-PRS effect on motor function explained by endophenotypes, we compared the variance of motor function explained by AD-PRS and that given each molecular phenotype estimated by relaimpo R package^52^. To infer the relationships among AD-PRS, endophenotypes, and a motor function, we used a Bayesian network (BN) framework^53,54^. Details were described in the Supplementary Methods.

## Results

### AD-PRS is associated with motor function in older adults

To investigate the effect of AD-PRS on motor functions, we focused on two motor phenotypes: global parkinsonism score and global motor score. We generated the AD-PRS based on GWAS summary statistics for Alzheimer’s dementia from IGAP^43^, each with varying *p* value criteria for SNP inclusion. We then tested the association of AD-PRSs with motor scores via linear regression. We set a significance threshold at *p* value of 0.001, as recommended^47^. The AD-PRS based on IGAP showed associations with both the global parkinsonism and global motor scores with an SNP inclusion threshold of *p* < 0.5 (Fig. 1b and Supplementary Table 4). To increase the robustness of our findings, we generated another AD-PRS based on GWAS for family history of Alzheimer’s dementia from CCACE^44^. As expected from the correlation between these AD-PRSs (Supplementary Fig. 1), the same association trends were observed for the AD-PRS based on the CCACE study (Fig. 1b). We also calculated the PD-PRSs^46^, to examine whether genetic risk for PD explains some variances of motor dysfunctions in older adults. However, the PD-PRSs were not associated with the motor scores, but moderately with the clinical diagnosis of Parkinson disease (PD-PRS with a SNP inclusion threshold of 0.5, odd ratio = 1.2, *p* = 0.0008) (Fig. 1b). Conversely, the AD-PRSs were not associated with the incidence of PD (Fig. 1b).

In further analyses, we examined whether the AD-PRS was differentially associated with the four parkinsonian signs used to construct global parkinsonism or the five motor abilities used to construct the global motor score. We used PRSs with a SNP inclusion threshold of 0.5 for the remaining analyses. The AD-PRSs showed strong associations with gait and rigidity domains in global parkinsonism score and dexterity and hand strength domain in global motor score, whereas tremor and balance ability assessed by stand tests were not associated with the AD-PRSs (Fig. 1c and Supplementary Table 5). These motor functions also showed significant (*p* < 0.05) or nearly significant associations with AD-PRS based on either IGAP, CCACE, or both in a participant group of Alzheimer’s dementia (*N* = 557). Conversely, these associations were not seen in a participant group of non-demented individuals (*N* = 1211) (Supplementary Table 6); this suggested that AD-PRS contributes to the motor impairment tied with dementia. Since the *APOE*/*TOMM40* locus contains the strongest genetic risk factor for Alzheimer’s dementia, we examined the effect of *APOE*/*TOMM40* on these associations by generating the AD-PRSs without *APOE*/*TOMM40* SNPs and also evaluating the independent contribution of *APOE*/*TOMM40* SNPs. The AD-PRSs without the *APOE*/*TOMM40* SNPs remained associated with the composite motor scores and domain scores to a similar extent as the AD-PRSs including *APOE*/*TOMM40* SNPs (Fig. 1c and Supplementary Table 5). *APOE*/*TOMM40* SNPs were moderately associated with the dexterity domain of the global motor score (*p* = 0.002) (Fig. 1c), but the overall impact on motor function was limited.

### Identifying the molecular mechanisms that link AD-PRS to motor impairment

**Fig. 2:**
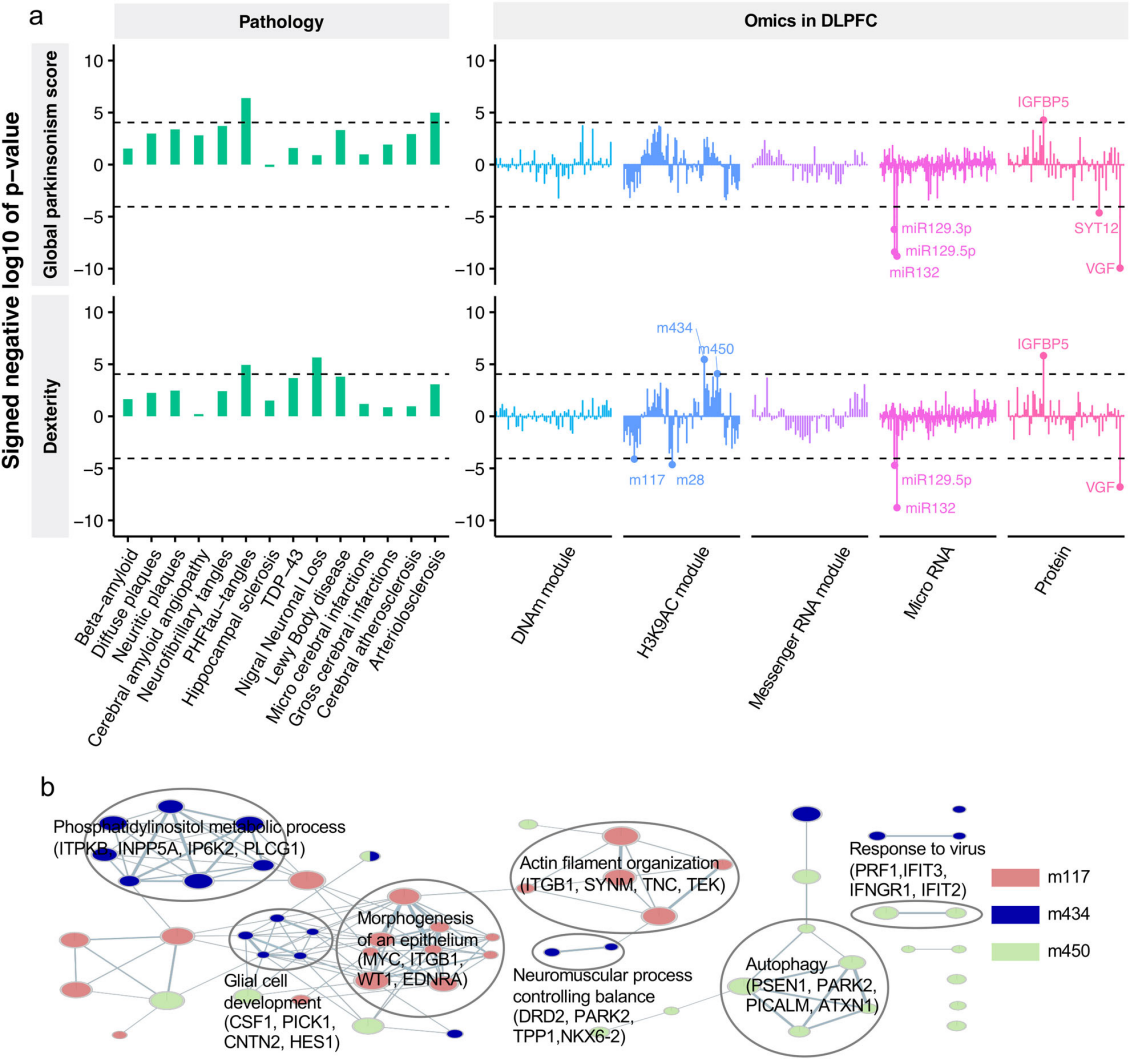
Neuropathological and molecular traits mediating the AD-PRS to motor impairment. **a** Associations of neuropathologies and molecular signatures with global parkinsonism score and dexterity. The sign of negative log 10 of *p* value indicates the direction of effect. **b** GO enrichment map for histone coacetylation modules. GO enrichment for histone coacetylation modules were conducted using the GREAT algorithm and the significant associations (FDR < 0.05) were visualized by EnrichmentMap

To aid in understanding the biological processes that explain AD-PRS mediation of motor impairment, we first screened the molecular phenotypes that correlate with the AD-PRS-associated motor functions. In this analysis, we used neuropathology and omics data from 552 individuals who had at least four out of five omics measurements from DLPFC (Supplementary Table 1). After aggregating the molecular data into covarying “modules,”^55^ this collection of data yielded 560 variables including 14 brain pathologies. In this test, we focused on the global parkinsonism score and dexterity, both of which remained strongly associated with AD-PRS in omics cohort (*p* < 1.0 × 10^–3^) (Supplementary Fig. 2). The associations of the 560 molecular variables with the two motor functions were examined by linear regression. We adopted a *p* value of 0.05/560 as a Bonferroni-corrected significance threshold (*p* < 8.9 × 10^–5^). In this comparison, we identified three brain pathologies (PHFtau-tangles, arteriolosclerosis, and nigral neuronal loss), four histone coacetylation modules (m28, m117, m434, and m450), three miRNAs (*miR-132*, *miR-129-5p*, and *miR-129-3p*), and three proteins (IGFBP5, VGF, and SYT12) that were associated with either global parkinsonism, dexterity, or both (*p* < 8.9 × 10^−5^) (Fig. 2a and Supplementary Table 7). To understand biological functions tied with histone coacetylation modules, we performed GO enrichment analysis based on genes located in the *cis*-regions of the histone acetylation peaks and found that three modules were enriched with at least one GO term (false discovery rate (FDR) <0.05) (Fig. 2b and Supplementary Table 8). For instance, m117 (287 peaks) was associated with epithelial morphogenesis (2.3-fold, *p* = 1.8 × 10^–5^) and actin filament organization (2.5-fold, *p* = 3.4 × 10^–5^); m434 (436 peaks) was enriched for phosphatidylinositol metabolic process (3.3-fold, *p* = 1.7 × 10^–6^), glial cell development (4.5-fold, p = 2.3 × 10^–6^), and neuromuscular process controlling balance (4.6-fold, p = 1.7 × 10^–5^); m450 (370 peaks) was involved in autophagy (2.6-fold, *p* = 3.0 × 10^–5^) and response to viruses (4.7-fold, *p* = 5.7 × 10^–6^). Interestingly, the association of m434 with neuromuscular processes controlling balance was supported by dopamine receptor D2 (*DRD2*) and parkin (*PARK2*), both of which are regarded as key molecules for parkinsonism.

**Fig. 3:**
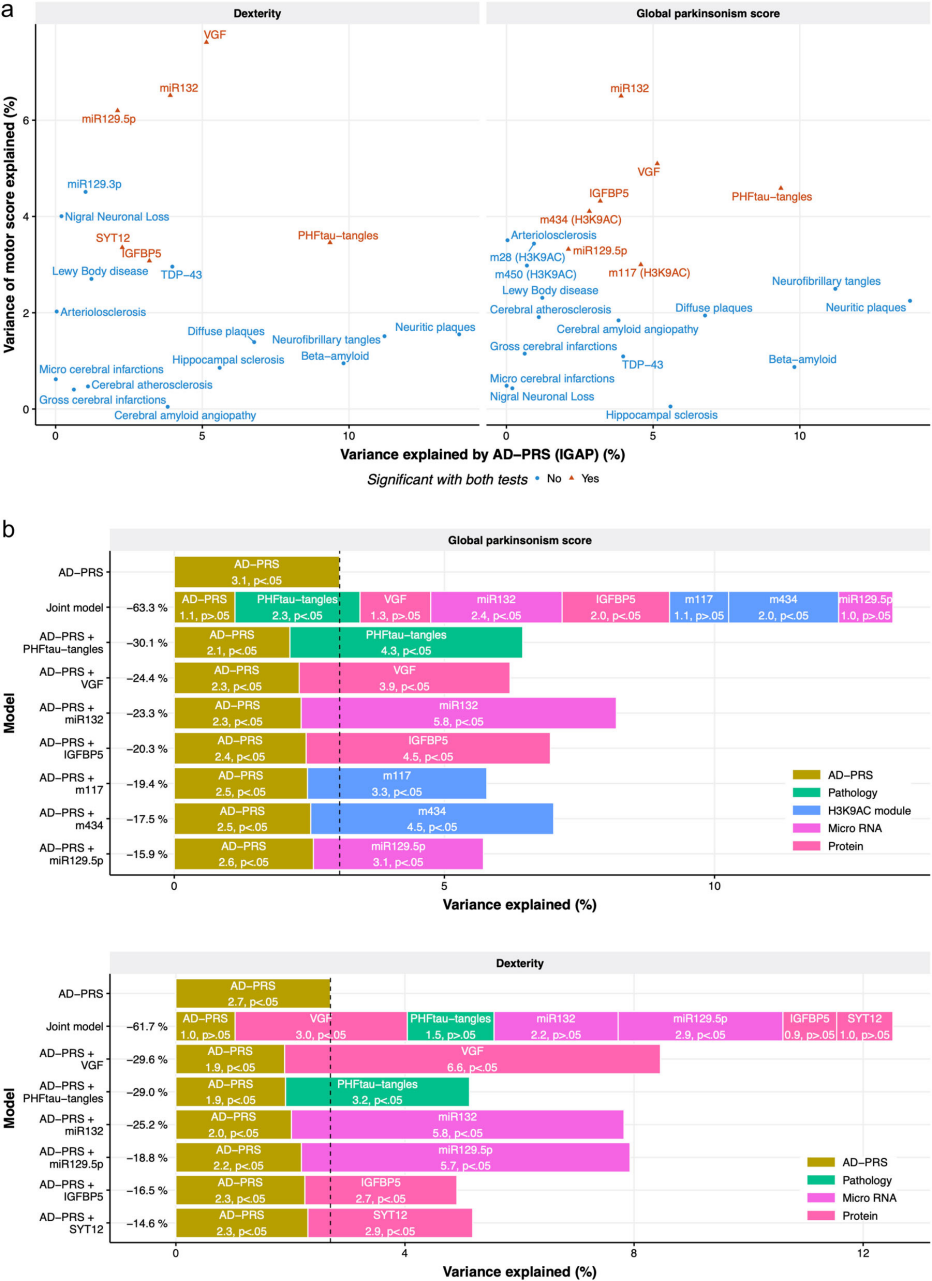
Molecular and neuropathological features that link AD-PRS and motor impairment. **a** Comparison between genetic and motor associations with neuropathologies and molecular signatures. Neuropathologies without associations with motor functions were also presented in this figure to contrast their genetic and motor associations. **b** The effect of the AD-PRS on motor functions explained by endophenotypes. The individuals who had the complete measurement for the variables were used (*n* = 480 for global parkinsonism score and *n* = 516 for dexterity)

Next, these 13 molecular phenotypes were further examined for their associations with the AD-PRS. As a result, one brain pathology, two histone coacetylation modules, two miRNAs, and three proteins were identified (*p* value after Bonferroni correction <0.05; nominal *p* < 0.0038) (Fig. 3a and Supplementary Table 9). The result suggests that the effect of the AD-PRS on motor functions could be explained by these molecular phenotypes. Interestingly, the molecular features associated with the AD-PRS did not always covary with motor function. For instance, the AD-PRS was associated strongly with β-amyloid burden (*p* = 5.5 × 10^–14^), but β-amyloid showed less association with motor abilities (*p* = 0.03 for global parkinsonism score and *p* = 0.02 for dexterity), while PHFtau tangles was associated with both the AD-PRS (*p* = 2.3 × 10^–13^) and motor abilities (*p* = 4.0 × 10^–7^ for global parkinsonism score and *p* = 1.1 × 10^–5^ for dexterity) (Fig. 3a).

**Fig. 4:**
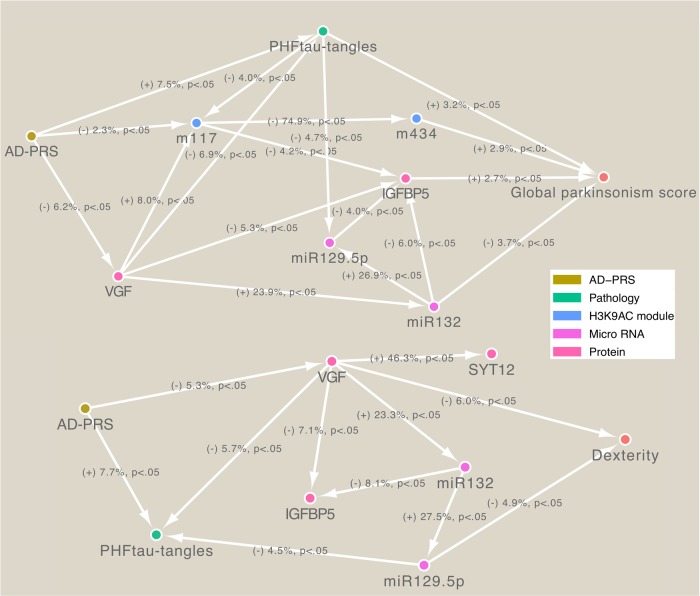
Bayesian network modeling of AD-PRS mediation to motor abilities. The individuals who had the complete measurement for the variables were used (*n* = 480 for global parkinsonism score and *n* = 516 for dexterity). A label of edge indicates the sign of the effect, variance explained, and *p* value calculated via linear regression

To gauge the magnitude of genetic effect mediated by molecular features, we contrasted the variance of motor abilities explained by the AD-PRS before and after controlling for each endophenotype. Each brain pathology and molecular hallmark explained 15–30% of the effects of AD-PRS on motor abilities (Fig. 3b) and together explained ~60% of the effects on the global parkinsonism score and dexterity. This suggests that the AD-PRS affects multiple molecular pathways that together lead to motor impairment. To further track the paths from AD-PRS to motor abilities, we inferred a BN among the AD-PRS, brain pathologies, omics, and motor functions (Fig. 4). Estimated BN structures suggested that the nodes for brain pathologies and omics formed interconnected regulatory networks and then mediate the AD-PRS’s effects on motor abilities through molecular cascades rather than independent paths. Further, the BN models indicated that direct upstream regulators for the global parkinsonism score were *miR-132*, PHFtau-tangles, m434, and IGFBP5, and those for dexterity were VGF and *miR-129-p5*. Thus, these molecular and pathological factors might be key drivers for motor impairment that are caused by the genetic risk for Alzheimer’s dementia.

## Discussion

A wide range of motor symptoms are associated with Alzheimer’s dementia and AD pathology^6,56–59^. However, it is unclear if these motor impairments in older adults are provoked by genetic factors for Alzheimer’s dementia. We found that the genetic risk for Alzheimer’s dementia (Fig. 1b) influenced motor functions in older adults, suggesting that cognitive and motor impairment share, at least in part, an underlying genetic architecture. The association was robust as they were observed with PRSs based on two separate GWASs for Alzheimer’s dementia (Fig. 1b). The PD-PRS was moderately associated with the clinical diagnosis of Parkinson disease, but it was not a strong predictor of global parkinsonism score or global motor score and its domains in the ROSMAP cohort. This might be because the parkinsonism score could be low in the patients with PD, as they may be treating such symptoms with PD therapies, and the PD-PRS was based on a subset of summary statistics of PD-GWAS that was the only panel publicly available. Thus, the result does not contradict the influence of PD genetics on motor function. The major genetic drivers for the associations between the AD-PRS and motor function originated from multiple loci below the genome-wide significance threshold (Fig. 1b), which agrees with the current understanding of the genetic architecture of Alzheimer’s dementia, where collective weak contributions explain the majority of genetic heritability^12^. This also suggests that the responsible genes for motor impairment may lie outside of genes supported by genome-wide significant loci.

Screening of potential biologic factors linking the genetic risk for Alzheimer’s dementia with motor impairment identified molecules and brain pathologies that explain the majority of the genetic effect. In particular, PHFtau-tangles, histone coacetylation module (m434), expression levels of *miR-132* and *miR-129-5p*, and protein abundance of VGF and IGFBP5 showed stronger effects on either global parkinsonism score or dexterity (Fig. 3b) and potentially formed regulatory cascades contributing to motor impairment (Fig. 4). PHFtau-tangles, *miR-132*, VGF, and IGFBP5 also explained the association of AD-PRS with cognitive decline^35^, suggesting these are involved in the common molecular mechanisms leading to cognitive decline and motor impairment in older adults. We also found that molecular features associated with AD-PRS were not always drivers of motor impairment. For instance, neuritic plaques showed a strong association with the AD-PRS but not with motor abilities (Fig. 3a). Thus, these results suggest that our integrative genetic approach extracted or prioritized sub-components of AD-PRS-associated molecular systems that are likely to be key regulators of motor abilities in older adults. Interestingly, *miR-132*, VGF, and PHFtau-tangles are reported to be dysregulated in brains of both Alzheimer’s dementia and PD patients^60–62^. Also, m434 was enriched for *cis*-regulatory region coding genes involved in neuromuscular processes controlling balance, such as dopamine receptor D2, parkin, and *NKX6-2* that are associated with PD^63^ or ataxia^64^. Thus, these molecular features might point to common mechanisms among these conditions that present with motor deficits.

Selected molecular and pathological features we identified may be prime targets for understanding the mechanisms of motor impairment in persons with Alzheimer’s dementia. Nonetheless, the study has several limitations. First, multi-omics data was restricted to the DLPFC region. It is possible that different and/or more robust associations would be found with omic data generated from regions more directly implicated in motor function, such as supplementary and primary motor cortex, basal ganglia, and substantia nigra, as well as spinal cord and muscle, the final effector of movement. In addition to brain, most participants in these studies have spinal cord, nerve and muscle tissue available for interrogation. Second, ROS and MAP are voluntary cohorts, and participants used in this study were highly educated and are primarily of European descent. Thus, the replication study by data from other, more diverse longitudinal cohorts is required to generalize these findings. However, the study also has many strengths in the integration of multiple types of omics data form the same individuals, along with comprehensive cognitive and motor evaluations. This allows us to perform an integrative analysis to untangle the molecular paths from genetic risk to motor impairment.

## Supplementary information

Supplementary Text and Figures

Supplementary Table 1

Supplementary Table 2

Supplementary Table 3

Supplementary Table 4

Supplementary Table 5

Supplementary Table 6

Supplementary Table 7

Supplementary Table 8

Supplementary Table 9
