# Supplementary material for: Genetic risk for Alzheimer’s dementia predicts motor deficits through multi-omic systems in older adults
Source: Transl Psychiatry. 2019 Oct 3;9:241. doi: 10.1038/s41398-019-0577-4 (PMC6776503; doi:10.1038/s41398-019-0577-4)
Supplement: Supplementary file 1 — Supplementary Text and Figures [file 41398_2019_577_MOESM1_ESM.pdf]

Genetic risk for Alzheimer's dementia predicts motor deficits through multi-omic systems in older adults

Authors: Shinya Tasaki PhD[1,2*], Chris Gaiteri PhD[1,2], Vladislav A. Petyuk PhD[3], Katherine D. Blizinsky PhD[1,2], Philip L. De Jager MD, PhD[4,5], Aron S. Buchman MD[1,2], David A. Bennett MD[1,2]

Affiliations:

[1] Rush Alzheimer's Disease Center, Rush University Medical Center, Chicago IL

[2] Department of Neurological Sciences, Rush University Medical Center, Chicago, IL, USA

[3] Biological Sciences Division, Pacific Northwest National Laboratory, Richland, WA, USA

[4] Center for Translational and Computational Neuroimmunology, Columbia University Medical Center, New York, NY, USA

[5] Cell Circuits Program, Broad Institute, Cambridge, MA, USA

[*] corresponding author

Supplementary Methods

Assessment of neuropathology indices

Tissue was dissected from eight brain regions to quantify the load of parenchymal deposition of β-amyloid by image analysis and the density of abnormally phosphorylated paired helical filament tau (PHFtau)-positive neurofibrillary tangles by stereology, as previously described[1–4]. Bielschowsky silver stain was used to visualize neuritic plaques, diffuse plaques, and neurofibrillary tangles in the frontal, temporal, parietal, and entorhinal cortex and the hippocampus. We created standardized scores for each plaque and tangle count in each cortical area. These scaled scores for each region were then averaged across the five regions. as previously described[2–5]. We performed square root transformed for diffuse plaques, neuritic plaques, neurofibrillary tangles, β-amyloid, and PHFtau-tangles. Nigral neuronal loss was assessed in the substantia nigra in the mid to rostral midbrain, near or at the exit of the third cranial nerve, using H&E stain and six micron sections using a dichotomous scale (absent or present)[6]. Lewy bodies were assessed in six regions using a monoclonal phosphorylated antibody to a-synuclein[7]. TDP-43 staging from amygdala to limbic and neocortical regions was determined using monoclonal TDP-43 antibody[8]. Hippocampal sclerosis was evaluated in a coronal section of the mid-hippocampus at the level of the lateral geniculate body and was reported as present if there was severe neuronal loss and gliosis in the CA1 sector, subiculum or both[9]. Chronic macroscopic and microinfarcts infarcts were recorded during gross examination and confirmed histologically[10]. Cerebral amyloid angiopathy (CAA) was assessed in four neocortical regions using immunohistochemistry[11]. β-amyloid depositions in meningeal and parenchymal vessels in each region were rated and averaged to obtain a summary CAA measure. Severity of atherosclerosis was graded by gross examination of vessels in the circle of Willis,

and arteriolosclerosis was graded on H&E stained sections of basal ganglia[12]. The complete list of brain pathologies assessed in this study is in Supplementary Table 2.

Omics measurements

Details on omics data processing were published previously[13]. Briefly, genotyping data was measured using DNA extracted from peripheral blood mononuclear cells or frozen brain tissue, and quality control steps were performed as described previously[13,14]. After the quality control steps, genotyping data included 7,159,943 SNPs in 2,093 subjects. DNA methylation data were generated using DNA extracted from DLPFC[15] and pre-processed as described previously[16]. The pre-processed data consisted of ~130,000 methylation loci in 533 subjects. Histone H3 acetylation on lysine 9 (H3K9AC) data was measured by Chromatin Immunoprecipitation (ChIP) assay using anti-H3K9AC mAb coupled with sequencing was performed in gray matter of DLPFC[17]. Quality control steps were described previously[13,17,18]. The pre-processed data consisted of 26,384 histone peaks in 516 subjects. RNAseq data was generated from DLPFC and quality control steps were performed as described previously[19,20]. The pre-processed data consisted of 13,484 genes in 432 subjects. To alleviate a large multiple testing burden, we followed the standard practice of reducing DNA methylation, histone acetylation, and gene expression to comethylated, coacetylated, and coexpressed modules[21], each of which was composed of variables with similar patterns of methylation, acetylation, or expression, as measured across all individuals. Using SpeakEasy[22], we identified 58 DNA comethylation modules, 80 histone coacetylation modules, and 49 coexpression modules[13]. The miRNA expression profiles were measured in DLPFC samples using the NanoString nCounter miRNA

expression assay and pre-processing steps were performed as described previously[13,23]. The pre-processed data consisted of 292 miRNAs in 543 subjects. Selected reaction monitoring (SRM) proteomics was performed using frozen DLPFC tissue for 67 proteins selected by the consortium members of Accelerating Medicines Partnership for Alzheimer's Disease (AMP-AD; https://www.synapse.org/#!Synapse:syn2580853)[24]. Pre-processing steps were described previously[13,24]. The pre-processed data consisted of 67 proteins in 527 subjects.

Gene ontology (GO) enrichment analysis for histone coacetylation modules

To examine whether histone coacetylation modules were involved in particular biological processes, we performed GO enrichment analysis. GO gene sets were downloaded from MSigDB v6.1[25,26]. The GREAT algorithm[27] was used for the enrichment analysis of *cis* regions of histone coacetylation modules with gene sets. The BSgenome.Hsapiens.UCSC.hg19 and TxDb.Hsapiens.UCSC.hg19.knownGene R packages were used for background information. The genomic region from 1,000 kb upstream of the transcriptional start site (TSS) to 1,000 kb downstream of the transcriptional end site (TES) was assigned for each gene in GO. If other genes were present within 1,000 kb upstream or downstream of the TSS or TES, respectively, of the gene of interest, the genomic region assigned for that gene was truncated at the point where the coding regions of other genes started. The genomic regions for all genes in each GO were then merged. Finally, a binomial test was used to evaluate whether the center locations of histone peaks in each module were enriched in the genomic region assigned to each GO. The Enrichment Map was utilized for visualization of the results [28].

Estimation of AD-PRS effect explained by endophenotypes

To evaluate the proportion of AD-PRS effect on motor function explained by endophenotypes, we compared the variance of motor function explained by AD-PRS and that given each molecular phenotype as follows:

First, the total variance of motor function was computed as the total sum of squares (SS),

$$SS_{total} = \sum_i (y_i - \bar{y})^2$$

where y_i is motor function for i individual, and $\bar{y}$ is an average of motor function. Then motor function was regressed with AD-PRS alone,

$$f^1(ADPRS) = a_1 + b_1 * ADPRS + \varepsilon_1$$

where a_1 is an intercept, b_1 is a coefficient for AD-PRS, and ε_1 is an error term. The SS for the residual of the first model was computed as

$$SS^1_{residual} = \sum_i (y_i - f^1_i)^2$$

and then the proportion of variance explained by AD-PRS was calculated as

$$PVE^1 = 1 - \frac{SS^1_{residual}}{SS_{total}}.$$

Next, motor function was regressed with AD-PRS and a mediator (M),

$$f^2(ADPRS, M) = a_1 + b_2 * ADPRS + c_2 * M + \varepsilon_2$$

where a_2 is an intercept, b_2 is a coefficient for AD-PRS, c_2 is a coefficient for a mediator, and ε_2 is an error term. The SS for the residual of the second model was computed as

$$SS^2_{residual} = \sum_i (y_i - f^2_i)^2$$

and then the proportion of variance explained by AD-PRS was calculated as

$$PVE^2_{ADPRS} = \left(1 - \frac{SS^2_{residual}}{SS_{total}}\right) * RI_{ADPRS}$$

The component RI_{ADPRS} is the relative contribution of AD-PRS to the variance explained by the second model and was calculated using the variance decomposition method proposed by Chevan and Sutherland[29].The method is implemented in relaimpo R package[30]. Lastly, the percent of AD-PRS effect explained by a mediator (PAEM) was computed as

$$PAEM = \frac{PVE^1 - PVE^2_{ADPRS}}{PVE^1} * 100.$$

Estimation of Bayesian network

To infer the relationships among AD-PRS, endophenotypes, and a motor function, we used a Bayesian network, which is a multivariate probabilistic model whose conditional independence relations can be represented by a directed acyclic graph (DAG) with *vertices* $V = (V_1, \dots, V_p)$, and *directed edges* $(i, j) \in E \subset V \times V$ (note that we use the notation i and V_i, interchangeably, to refer to a node). A vertex j in a DAG G corresponds to a random variable X_j in the Bayesian network. Assuming the local directed Markov property, each variable is independent of its non-descendant variables conditional on its parent variables. Thus, the state of X_j can be determined only by the state of parent variables, which is formally expressed by the conditional probability, $P(X_j \mid X_{G_j})$, where X_j state occurs under given parents' state X_{G_j}. Therefore, the probability where observed data, X, is generated from a given DAG G can be factored as $P(X \mid G) = \prod_{j=1}^{p} P(X_j \mid X_{G_j})$, where $X = (X_1, \dots, X_p)^T$, G_j is the set of parents of j, and $X_{G_j} = \{X_i : i \in G_j\}$. To learn the DAG structure, which is the process of finding G with high $P(X \mid G)$, we used a Markov chain Monte Carlo (MCMC) method to sample DAGs based on the posterior distribution of DAG structures

$$P(G \mid X) = \frac{P(X \mid G)P(G)}{\sum_{G \in \mathcal{G}} P(X \mid G)P(G)},$$

where $P(G)$ is a prior on the network structure G, and $\mathcal{G}$ represents the space of all DAGs with p vertices. The MCMC sampling allows us to obtain ensembles of DAGs with high $P(X \mid G)$ and avoid overfitting to the data. To utilize genetic information as a clue to infer the directions of other edges, we restricted a direction of edges so that AD-PRS can have only out-going edges to other nodes. We did not pose any restrictions for non-genetic nodes. We ran 300,000 steps of MCMC sampling using the REV algorithm[31] and discarded the first 2,400 steps as a burn-in. Then, edge frequencies in the sampled networks were counted, and a consensus network was generated by taking the regulation that presented most frequently among the three possible: node1 regulates node2, node2 regulates node1, and node1 is independent of node2. The detailed implementation of learning network structure based on systems genetics data is described elsewhere[32]. Given the estimated network structure, each variable was regressed with its parent variables and obtained variance explained by each parent variable and p-value associated with it.

Supplementary references

1. Bennett DA, Wilson RS, Boyle PA, Buchman AS, Schneider JA. Relation of neuropathology to cognition in persons without cognitive impairment. Ann Neurol 2012; 72: 599–609.
2. Bennett DA, Schneider JA, Arvanitakis Z, Kelly JF, Aggarwal NT, Shah RC, et al. Neuropathology of older persons without cognitive impairment from two community-based studies. Neurology 2006; 66: 1837–1844.
3. Boyle PA, Wilson RS, Yu L, Barr AM, Honer WG, Schneider JA, et al. Much of late life cognitive decline is not due to common neurodegenerative pathologies. Ann Neurol 2013; 74: 478–489.
4. Schneider JA, Arvanitakis Z, Yu L, Boyle PA, Leurgans SE, Bennett DA. Cognitive impairment, decline and fluctuations in older community-dwelling subjects with Lewy bodies. Brain 2012; 135: 3005–3014.
5. Bennett DA, Schneider JA, Arvanitakis Z, Wilson RS. Overview and findings from the religious orders study. Curr Alzheimer Res 2012; 9: 628–645.
6. Buchman AS, Shulman JM, Nag S, Leurgans SE, Arnold SE, Morris MC, et al. Nigral pathology and parkinsonian signs in elders without Parkinson disease. Ann Neurol 2012; 71: 258–266.
7. Wilson RS, Yu L, Schneider JA, Arnold SE, Buchman AS, Bennett DA. Lewy bodies and olfactory dysfunction in old age. Chem Senses 2011; 36: 367–373.
8. Yu L, De Jager PL, Yang J, Trojanowski JQ, Bennett DA, Schneider JA. The

TMEM106B locus and TDP-43 pathology in older persons without FTLD. Neurology 2015; 84: 927–934.
9. Nag S, Yu L, Capuano AW, Wilson RS, Leurgans SE, Bennett DA, et al. Hippocampal sclerosis and TDP-43 pathology in aging and Alzheimer disease. Ann Neurol 2015; 77: 942–952.
10. Schneider JA, Bienias JL, Wilson RS, Berry-Kravis E, Evans DA, Bennett DA. The apolipoprotein E epsilon4 allele increases the odds of chronic cerebral infarction [corrected] detected at autopsy in older persons. Stroke 2005; 36: 954–959.
11. Yu L, Boyle PA, Nag S, Leurgans S, Buchman AS, Wilson RS, et al. APOE and cerebral amyloid angiopathy in community-dwelling older persons. Neurobiol Aging 2015; 36: 2946–2953.
12. Arvanitakis Z, Capuano AW, Leurgans SE, Bennett DA, Schneider JA. Relation of cerebral vessel disease to Alzheimer's disease dementia and cognitive function in elderly people: a cross-sectional study. Lancet Neurol 2016; 15: 934–943.
13. Tasaki S, Gaiteri C, Mostafavi S, De Jager PL, Bennett DA. The Molecular and Neuropathological Consequences of Genetic Risk for Alzheimer's Dementia. Front Neurosci 2018; 12: 699.
14. Shulman JM, Chen K, Keenan BT, Chibnik LB, Fleisher A, Thiyyagura P, et al. Genetic susceptibility for Alzheimer disease neuritic plaque pathology. JAMA Neurol 2013; 70: 1150–1157.
15. De Jager PL, Srivastava G, Lunnon K, Burgess J, Schalkwyk LC, Yu L, et al. Alzheimer's disease: early alterations in brain DNA methylation at ANK1, BIN1, RHBDF2 and other loci. Nat Neurosci 2014; 17: 1156–1163.
16. Gaiteri C, Dawe R, Mostafavi S, Blizinsky KD, Tasaki S, Komashko V, et al. Gene expression and DNA methylation are extensively coordinated with MRI-based brain microstructural characteristics. Brain Imaging Behav 2018;
17. Klein H-U, McCabe C, Gjoneska E, Sullivan SE, Kaskow BJ, Tang A, et al. Epigenome-wide study uncovers tau pathology-driven changes of chromatin organization in the aging human brain. bioRxiv 2018; 273789.
18. Tasaki S, Gaiteri C, Mostafavi S, Yu L, Wang Y, De Jager PL, et al. Multi-omic Directed Networks Describe Features of Gene Regulation in Aged Brains and Expand the Set of Genes Driving Cognitive Decline. Front Genet 2018; 9: 294.
19. Mostafavi S, Gaiteri C, Sullivan SE, White CC, Tasaki S, Xu J, et al. A molecular network of the aging human brain provides insights into the pathology and cognitive decline of Alzheimer's disease. Nat Neurosci 2018; 21: 811–819.
20. Ng B, White CC, Klein H-U, Sieberts SK, McCabe C, Patrick E, et al. An xQTL map integrates the genetic architecture of the human brain's transcriptome and epigenome. Nat Neurosci 2017; 20: 1418–1426.
21. Zhang B, Horvath S. A general framework for weighted gene co-expression network analysis. Stat Appl Genet Mol Biol 2005; 4: Article17.
22. Gaiteri C, Chen M, Szymanski B, Kuzmin K, Xie J, Lee C, et al. Identifying robust communities and multi-community nodes by combining top-down and bottom-up approaches to clustering. Sci Rep 2015; 5: 16361.
23. Patrick E, Rajagopal S, Wong H-KA, McCabe C, Xu J, Tang A, et al. Dissecting the role of non-coding RNAs in the accumulation of amyloid and tau neuropathologies in Alzheimer's disease. Mol Neurodegener 2017; 12: 51.
24. Yu L, Petyuk VA, Gaiteri C, Mostafavi S, Young-Pearse T, Shah RC, et al. Targeted brain proteomics uncover multiple pathways to Alzheimer's dementia. Ann Neurol 2018;

25. Subramanian A, Tamayo P, Mootha VK, Mukherjee S, Ebert BL, Gillette MA, et al. Gene set enrichment analysis: a knowledge-based approach for interpreting genome-wide expression profiles. Proc Natl Acad Sci U S A 2005; 102: 15545–15550.
26. Liberzon A, Birger C, Thorvaldsdóttir H, Ghandi M, Mesirov JP, Tamayo P. The Molecular Signatures Database (MSigDB) hallmark gene set collection. Cell Syst 2015; 1: 417–425.
27. McLean CY, Bristor D, Hiller M, Clarke SL, Schaar BT, Lowe CB, et al. GREAT improves functional interpretation of cis-regulatory regions. Nat Biotechnol 2010; 28: 495–501.
28. Merico D, Isserlin R, Stueker O, Emili A, Bader GD. Enrichment map: a network-based method for gene-set enrichment visualization and interpretation. PLoS One 2010; 5: e13984.
29. Chevan A, Sutherland M. Hierarchical Partitioning. Am Stat 1991; 45: 90.
30. Grömping U. Relative Importance for Linear Regression in R : The Package relaimpo. J Stat Softw 2006; 17: 1–27.
31. Grzegorczyk M, Husmeier D. Improving the structure MCMC sampler for Bayesian networks by introducing a new edge reversal move. Mach Learn 2008; 71: 265–305.
32. Tasaki S, Sauerwine B, Hoff B, Toyoshiba H, Gaiteri C, Chaibub Neto E. Bayesian network reconstruction using systems genetics data: comparison of MCMC methods. Genetics 2015; 199: 973–989.

Supplementary Figures

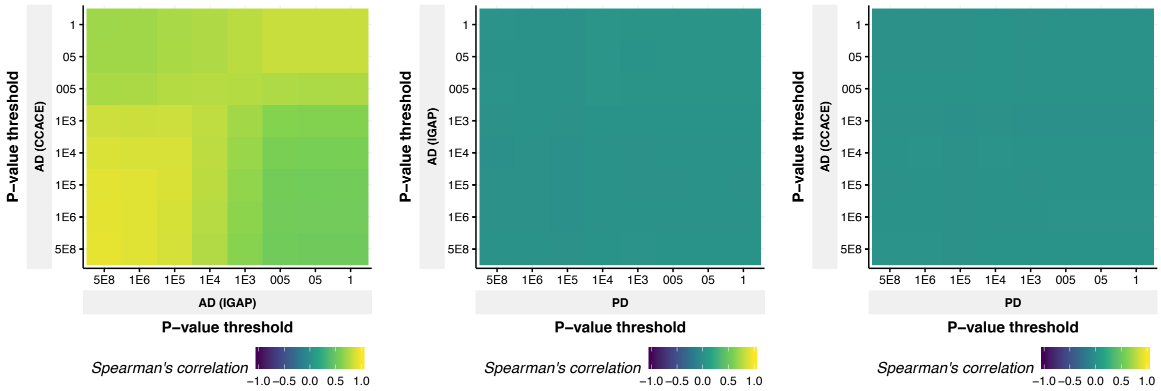

Supplementary Figure 1. Correlation between PRSs from different GWAS panels.

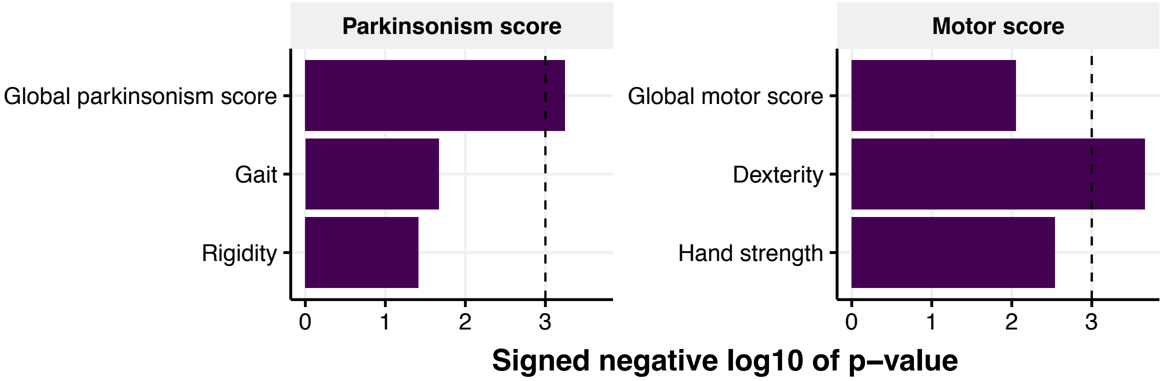

Supplementary Figure 2. Association of motor phenotypes with the AD-PRS in omics cohort.
